# A tRNA methyltransferase paralog is important for ribosome stability and cell division in *Trypanosoma brucei*

**DOI:** 10.1038/srep21438

**Published:** 2016-02-18

**Authors:** Ian M. C. Fleming, Zdeněk Paris, Kirk W. Gaston, R. Balakrishnan, Kurt Fredrick, Mary Anne T. Rubio, Juan D. Alfonzo

**Affiliations:** 1 Department of Microbiology and Center for RNA Biology, The Ohio State University, Columbus, Ohio 43210, USA; 2 Rieveschl Laboratories for Mass Spectrometry, Department of Chemistry, University of Cincinnati, Cincinnati, Ohio 45221, USA; 3 Ohio State Biochemistry Program, The Ohio State University, Columbus, Ohio 43210, USA f

## Abstract

Most eukaryotic ribosomes contain 26/28S, 5S, and 5.8S large subunit ribosomal RNAs (LSU rRNAs) in addition to the 18S rRNA of the small subunit (SSU rRNA). However, in kinetoplastids, a group of organisms that include medically important members of the genus *Trypanosoma* and *Leishmania*, the 26/28S large subunit ribosomal RNA is uniquely composed of 6 rRNA fragments. In addition, recent studies have shown the presence of expansion segments in the large ribosomal subunit (60S) of *Trypanosoma brucei*. Given these differences in structure, processing and assembly, *T. brucei* ribosomes may require biogenesis factors not found in other organisms. Here, we show that one of two putative 3-methylcytidine methyltransferases, TbMTase37 (a homolog of human methyltransferase-like 6, METTL6), is important for ribosome stability in *T. brucei*. TbMTase37 localizes to the nucleolus and depletion of the protein results in accumulation of ribosomal particles lacking srRNA 4 and reduced levels of polysome associated ribosomes. We also find that TbMTase37 plays a role in cytokinesis, as loss of the protein leads to multi-flagellated and multi-nucleated cells.

Eukaryotic ribosomes are comprised of a small 40S and a large 60S subunit; generally, these contain 4 rRNAs (18S, 28S, 5S and 5.8S) and more than 70 ribosomal proteins. While this generic textbook version of the cytoplasmic ribosome holds for many organisms, variations on this theme exist. For instance, the 28S rRNA (large subunit ribosomal RNA, LSU rRNA) is nicked into 2 fragments in some vertebrates, insects and protozoans^1^,^2^. In a subset of protists the LSU rRNA may be further fragmented as in the case of *Euglena gracilis* where the 28S rRNA is fragmented into 13 pieces or *Crithidia fasciculata* which possess a 4-part 28S rRNA^3^,^4^. Just as unusual is the situation in *Trypanosoma brucei* where in addition to a canonical 5S and 5.8S rRNA, the LSU 26/28S rRNA is split into 6 fragments of sizes 1827 (LSUα), 1485(LSUβ), 215 (srRNA 1, formerly SR1 or M1), 183 (srRNA 2, formerly SR2 or M2), 136 (srRNA 4, formerly SR4 or M4), and 78 nucleotides (srRNA 3, formerly SR6 or M6)^5^. A recent cryo-electron microscopy study showed that the *T. brucei* ribosome exhibits considerable structural similarly to the 80S ribosome of yeast, although several novel features stand out. These include 4 unique inter-subunit bridges, several additional proteins, lengthening and/or elaboration of various rRNA expansion segments, numerous protein extensions and a kinetoplastid-specific domain (KSD)^6^. Interestingly, one of these inter-subunit bridges is formed by srRNA 4 leading to the suggestion that fragmentation may have occurred to accommodate unique structural extensions created by the KSD^6^. With this added ribosome complexity, it is reasonable to expect the existence of novel protein factors, perhaps found exclusively in trypanosomes, that facilitate processing, assembly or contribute to stability. Indeed, several groups have reported proteins unique to kinetoplastids that are essential for ribosome stability and/or biogenesis^7^,^8^,^9^,^10^,^11^,^12^.

Ribosome biogenesis in trypanosomes starts with the synthesis of the various precursor rRNAs. RNA polymerase III transcribes 5S rRNA in the nucleoplasm while the remaining rRNAs are transcribed as a single polycistronic transcript in the nucleolus by RNA polymerase I. This transcript includes the 18S rRNA of the SSU and six of the seven parts of the LSU rRNA. This polycistronic RNA is matured by a series of cleavage and post-transcriptional modification steps involving small nucleolar RNAs, various endo- and exonucleases and several modification enzymes. The ribosomal particles are assembled by the binding of ribosomal proteins and concomitant folding of rRNA, presumably via multiple parallel pathways^13^,^14^. The biogenesis process is regulated and its efficiency depends on the correct stoichiometry of mature rRNAs and ribosomal proteins. Although rRNA transcription and the initial stages of ribosome biogenesis occur in the nucleolus, subsequent steps occur in the nucleus and the final steps take place in the cytoplasm. Detailed 80S ribosome biogenesis studies have been conducted in a few organisms including mammals, *Xenopus* and most extensively in the yeast *Saccharomyces cerevisiae*. These studies suggest that hundreds of proteins are involved in the biogenesis process to direct pre-rRNA processing, modify rRNA, shuttle assembling particles or chaperone assembly. Among these, only a subset is conserved across the eukaryotes, suggesting marked differences between organisms.

Because protein synthesis plays a central role in cell growth, nucleolar function and ribosome stability are intimately coupled with cell-cycle control in many organisms^15^. In the present study we have identified TbMTase37; a protein evolutionary related to a group of methyltransferases that includes yeast Trm140 (the tRNA m^3^C methyltransferase) and human METTL6 (a methyltransferase of unknown target). Depletion of TbMTase37 leads ribosome instability, the specific degradation of srRNA 4 in 60S subunits, and reduction in the number of polysome-associated ribosomes. Reduction in TbMTase37 levels also causes a defect in cytokinesis, suggesting a link between ribosome stability and/or function and cell division in *T. brucei*.

## Results

### Phylogenetic analysis of TbTrm140 and TbMTase37

**Figure 1:**
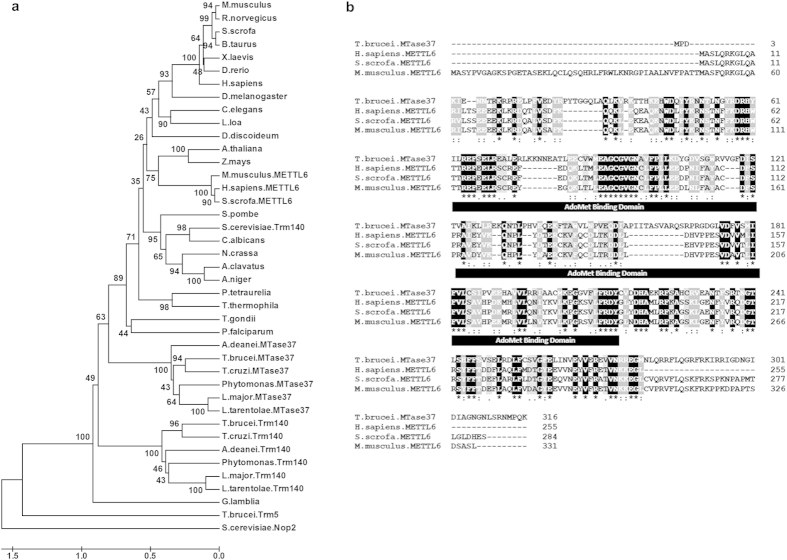
Two paralogous proteins in *T. brucei* methyltransferases belong to a family of methyltransferases that includes mammalian METTL6. (**a**) A UPGMA tree was generated following multiple sequence alignment of *S. cerevisiae* TRM140 (NP_014882.4) with orthologous sequences from other organisms using MEGA6. Multiple sequence alignment was accomplished with the MUSCLE algorithm. The UPGMA tree was generated from the alignment with default parameters and 10,000 bootstrap replicates. Bootstrap values are displayed as percentages at each tree node. (**b**) Sequence alignment of the two *T. brucei* paralogs (TbTRM140 and TbMTase37) with mammalian methyltransferase-like 6 (METTL6). Black boxes (*) indicate identical amino acids shared among the sequences, while amino acids highlighted in gray (: or .) denote conservative changes. The putative SAM-binding domain is indicated by the black filled rectangle labeled “AdoMet Binding Domain” and it is conserved in all four proteins.

We used the amino acid sequence of the m^3^C methylase (Trm140) from yeast^16^,^17^ as the query in Protein BLAST^18^. This revealed the presence of two Trm140 paralogs in *T. brucei*, which we term TbTrm140 (Tb927.10.1800) and TbMTase37 (Tb927.9.11750) (Fig. 1a). These sequences were used to generate a UPGMA (Unweighted Pair Group Method with Arithmetic Mean) tree that revealed polyphyletic relationships between m^3^C methyltransferases from numerous organisms including tRNA methyltransferase TRM140 from yeast and *T. brucei*, along with METTL6 of higher eukaryotes and MTase37 from kinetoplastids. For this analysis, two proteins that belong to the RNA methyltransferase family, but are unrelated to m^3^C formation, were used as outgroups. One of these, *T. brucei* TRM5, is a *bona fide* 1-methylguanosine tRNA methyltransferase for G_37_ of seven tRNAs^19^. This phylogenetic tree suggests that a gene-duplication event gave rise to TbTrm140 and TbMTase37 (Fig. 1a). Both candidate proteins contain a predicted and conserved C-terminal S-adenosyl-methionine (SAM) binding domain and all the key residues important for methylation by the yeast enzyme (Fig. 1b). The only major difference between these proteins is the absence of the N-terminal actin-binding domain found in *S. cerevisiae*; however, this domain is dispensable for methylation activity in yeast^16^,^17^. We have established that one of the genes (Tb927.10.1800) encodes the authentic TbTrm140 homolog responsible for tRNA methylation in *T. brucei* (M. Rubio, I. Fleming, K. Anderson, J.D. Alfonzo, unpublished data). However, no tRNA methylation activity could be ascribed to the second paralog either *in vitro* or *in vivo*. Notably, one of the homologs of TbMTase37 is the human METTL6 (methyltransferase-like protein 6), which despite having no known function has been reported as one of the drivers of cell proliferation in a sub-class of breast cancers^20^.

### A Trm140 methyltransferase paralog is important for cytokinesis in *T. brucei*

**Figure 2:**
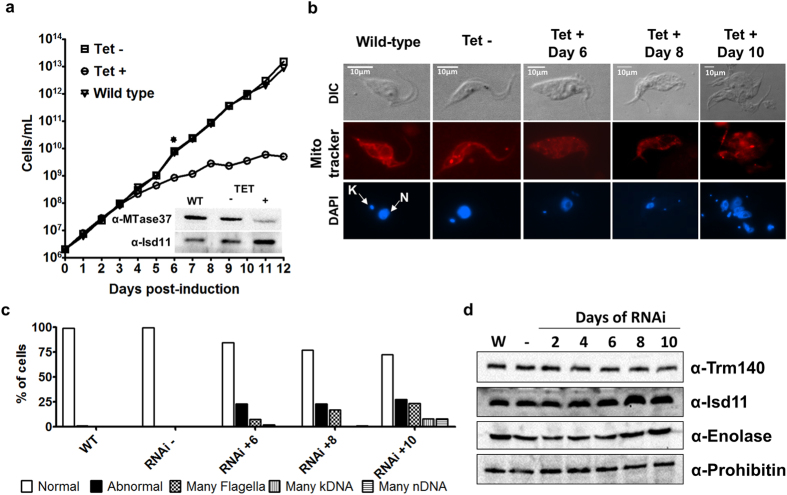
TbMTase37 is essential for normal cell division in T. brucei. (**a**) Growth curve of wild type, uninduced (Tet −) and induced (Tet + ) cell lines. Tet − and Tet + are T. brucei 29-13 cells transformed with the tetracycline-inducible MTase37 RNAi vector, integrated into genomic. RNAi was induced by tetracycline addition to growth media and monitored every 24h. Six days post-induction (indicated by  *) total protein extracts of each cell line were prepared. Western blots were performed using purified anti-TbMTase37 rabbit polyclonal antibodies. Protein extracts from equal number of cells of wild type, Tet −, and Tet + were loaded per lane. The inset shows a representative Western blot where “α-TbMTase37” and “α-Isd11” refer to separate blots of the same membrane performed using antibodies against each protein. The Isd11 protein was used as a loading control. (**b**) Immunofluorescence to monitor changes in cellular morphology, mitochondrial shape and cellular DNA. Wildtype, Tet − and Tet + cells collected at 6, 8, and 10 days post-induction are shown. DIC imaging was used to visualize flagellum and cell size. All cells were stained with MitoTracker (red) and DAPI (blue) to visualize mitochondrial shape and DNA content of either nuclei (N) or mitochondrial kDNA (K), respectively. Scale bars (white lines) represent 10μm. (**c**) Characterization of cell-division structural signals. For each cell-line and time-point in B, at least 100 cells were scored for number of flagella (F), kDNA (K), and nuclear DNA (N) to assess percentage of “abnormal” cells which did not follow typical cell-division progression of 1F:1K:1N, 2F:1K:1N, 2F:2K:1N, 2F:2K:2N and into 2 cells. Histogram bars are classified first as normal or abnormal and further by the structure(s) found to be abnormal in typical cell-division progression. (**d**) Western blots were performed using purified rabbit polyclonal antibodies against several T. brucei proteins (Trm140, Isd11, Enolase and Prohibitin) as gauge for translational defects. Protein extracts were generated from an equal number of wild type, Tet −, and Tet + cells at 2, 4, 6, 8 or 10 days of induction and equal volumes were loaded in the gel.

To explore further the potential biological function of TbMTase37, we placed a portion of the coding sequence of TbMTase37 in the tetracycline inducible RNAi vector p2T7-177 and monitored growth of induced, un-induced, and wild type cells^21^. Upon tetracycline addition, a pronounced slow-growth phenotype was evident 6 days post-induction compared to wild type and uninduced cells (Fig. 2a). To corroborate the down-regulation of TbMTase37, protein lysates were taken 6 days post-induction and analyzed by Western blotting using a TbMTase37-specific antibody raised against a recombinant version of TbMTase37 purified from *E. coli*. This experiment confirmed the reduction of TbMTase37 protein levels compared to the uninduced control (Fig. 2a, inset). As it typical of RNAi, our uninduced control lysate exhibits a decrease in MTase37 levels when compared to wild-type cells of about 50% and at this degree of depletion cells do not yet exhibit a slow growth phenotype^22^,^23^. Notably, upon RNAi induction and concomitantly with the onset and progression of the observed growth phenotype, cells showed abnormal shape and size, indicative of a cell division defect. Cells were analyzed by fluorescent microscopy to visualize flagellar structures, DNA content (nuclear and mitochondrial), cell shape, and mitochondrial integrity (Fig. 2b). Our results revealed a defect in cytokinesis typified by multiple flagella, nuclei and mitochondrial DNA (kDNA, kinetoplast DNA) per cell, as well as loss of the mitochondrial reticulation characteristic of *T. brucei*. That these “abnormal” cells were alive was established by staining with the membrane potential-dependent dye MitoTracker. By 10 days post-induction at least 25% of cells showed the “abnormal” phenotype with respect to cell shape and intracellular structure (Fig. 2c)^24^. To estimate the translation of a set of unrelated proteins along the course of RNAi protein lysates were prepared at 2 days intervals starting from the beginning of induction and analyzed by Western blotting. Using antibodies specific to four proteins (Trm140, Isd11, Enolase and Prohibitin) we found that protein levels do not differ after RNAi (Fig. 2d). Importantly, this Western blot analysis is not a global analysis of translation which will be our focus in future studies. These results indicate that TbMTase37 contributes in some way to cytokinesis in *T. brucei*.

### TbMTase37 is a nucleolar protein

**Figure 3:**
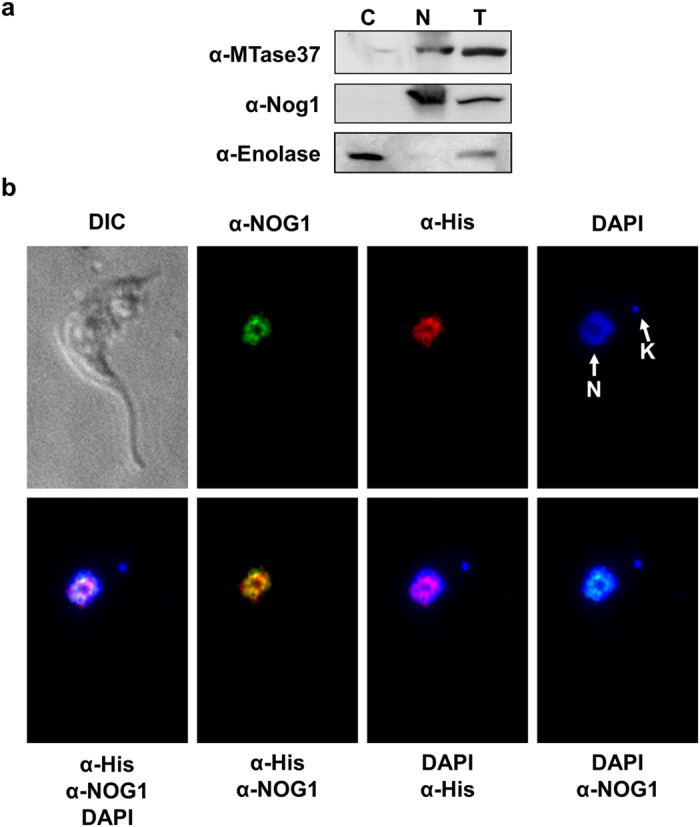
TbMTase37 is a nucleolar protein. (**a**) Western blot analysis was performed on total, nuclear (N) and cytoplasmic (C) protein fractions using polyclonal rabbit antibodies against TbMTase37, Nog1 (a nuclear/nucleolar marker) and Enolase (cytoplasmic marker). Anti-Enolase antibody (α-Enolase) was used as a control for cytoplasmic contamination in the nuclear fractions and anti-Nog1 antibody (α-Nog1) was used as a control for nuclear contamination of cytoplasmic fraction. Blots were generated with equal volumes of the same lysate on different gels and cropped panels represent the signal observed at the expected size of each protein. (**b**) Immunofluorescent localization using antibodies against NOG1 (α-NOG1) and anti-His antibody (α-His) to detect the His-tagged TbMTase37. DAPI (blue) was used to detect the nuclear (N) and mitochondrial kDNA (K). Fluorescent-labeled secondary antibodies were used to detect TbMTase37 (red) and NOG1 (green). DIC represents light microscope view of a single cell. Merged images are superimpositions of each single combination or all three channels together performed with ImageJ (NIH). Yellow fluorescence is indicative of co-localization of NOG1 and TbMTase37.

To further define the role of TbMTase37 in cell function, we used cell fractionation followed by Western blot analysis, in addition to immunofluorescence microscopy, to determine the intracellular localization of this protein. Western analysis of *T. brucei* sub-cellular fractions (total, nuclear, and cytoplasmic) showed that TbMTase37 is present in the nuclear fraction. In these experiments, antibodies against enolase and Nog1 were used as cytoplasmic and nuclear markers, respectively, and confirmed the purity of the nuclear fraction (Fig. 3a)^10^. The gene encoding TbMTase37 was then cloned into the *T. brucei* tetracycline inducible expression vector pLEW79, which places epitope-tags (Myc, His and Protein A) at the C-terminus of the protein. The resulting construct, pLew79-TbMTase37-MHA, was transfected into *T. brucei* 29-13 cells, and protein production was induced by addition of tetracycline. Immunofluorescence experiments with anti-His antibodies showed that TbMTase37 was restricted to a portion of the nucleus, suggesting nucleolar localization (Fig. 3b). Experiments performed using antibodies against the nucleolar marker protein NOG1 showed co-localization of the two proteins (Fig. 3b), confirming the nucleolar localization of TbMTase37^10^. Ideally, we would have performed the co-localization experiment with the anti-TbMTase37 antibody used in the Western blot experiment; however, the fact that both anti-TbMTase37 and anti-Nog1 are rabbit antibodies precluded such analysis. To rule out the possibility that the observed fluorescence with our anti-His antibody is non-specific, we also performed similar experiments with wild-type cells not expressing the His-tagged TbMTase37 (Fig. S2). This experiment showed no detectable fluorescence in the absence of the epitope-tagged TbMTase37 confirming the specificity of antibody binding. Taken together, these experiments provide strong evidence for the nuclear localization of TbMTase37 and more specifically to the nucleolus.

### TbMTase37 depletion leads to reduction in polysomes

**Figure 4:**
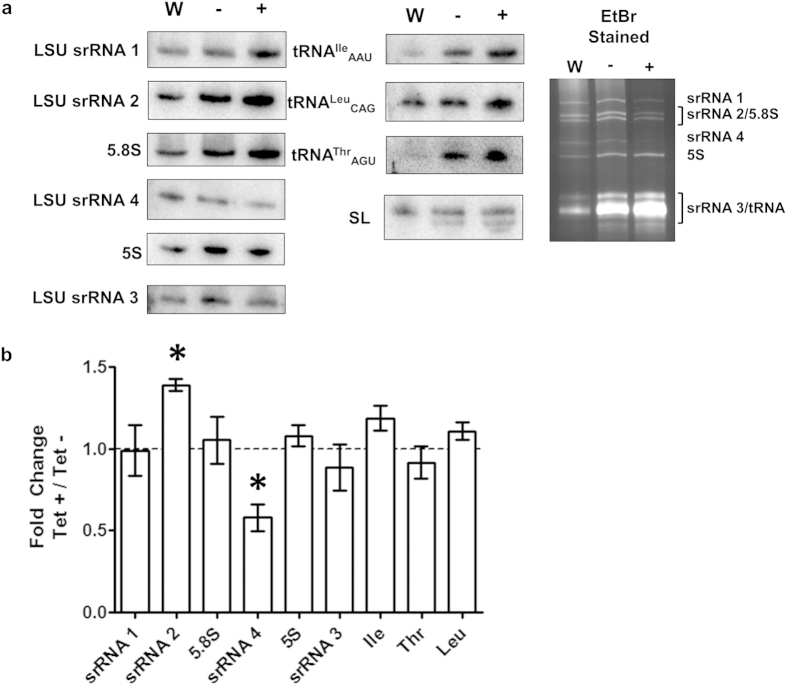
TbMTase37 RNAi affects steady-state RNA levels. (**a**) Total cellular RNA from wild type (W), uninduced (−) and induced (+) cells was analyzed by Northern blot with radioactive oligonucleotide probes specific for each small rRNA species and a subset of cellular tRNAs were used to detect changes in steady-state RNA levels after RNAi induction. Cytoplasmic spliced leader RNA (SL RNA) was used as a normalization control, where changes in RNA levels were calculated by normalization of wild type and induced signals to SL. Cropped panels represent the radioactive signal present at the expected RNA sizes and is also the area used in quantification. The ethidium bromide stained full-length gel (rightmost panel) shows equal RNA loading with equal A_260_ units in each lane. (**b**) The normalized ratio of induced signal was divided by the normalized wild type signal and graphed as “Fold Change”. Each bar represents an average of 3 independent gels and blotting experiments with error bars representing standard error of mean. Statistical analysis was performed using a one-sample t-test. An asterisk (*) represents a significant (P < 0.05) difference from 1.0 (no change, dashed line).

**Figure 5:**
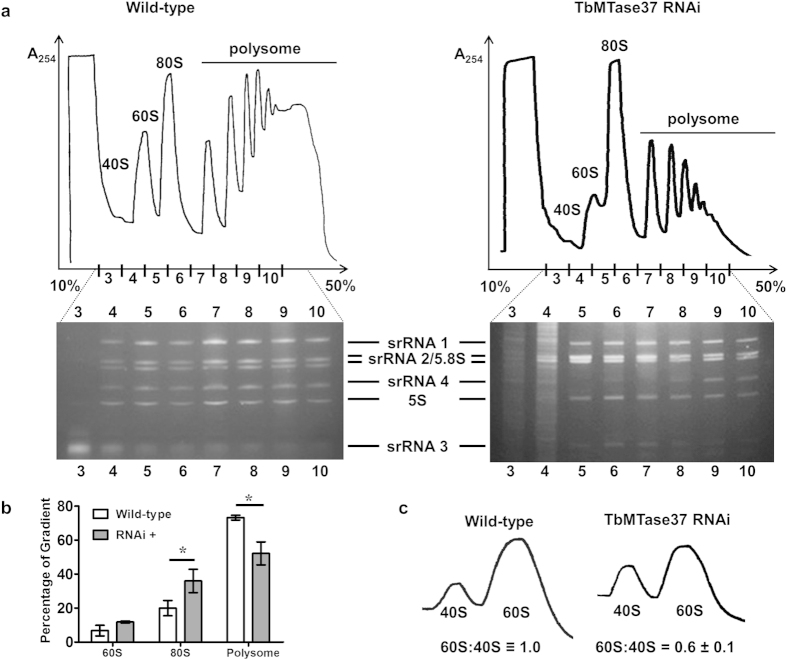
Down-regulation of TbMTase37 leads to reduction in Polysome-associated ribosomes and 60S subunit levels. (**a**) *T. brucei* lysates from wild type and RNAi cells were separated by sucrose gradient centrifugation (10–50% sucrose is shown) and fractions monitored by A_254_. Peaks corresponding to 40S, 60S, 80S and polysome-associated ribosome are as indicated. Lanes correspond to the fraction number indicated and the identity of the 6 small rRNA bands in the gel are as indicated. Gradients and gels were loaded with equal A_260_ units. (**b**) The area under polysome traces was quantified for wild-type and MTase37 RNAi polysomes experiments. Quantification was performed using the Measure feature of ImageJ (NIH). A baseline was first drawn across the bottom of polysome traces and then peaks were extrapolated down to the baseline. The area under the trace for 60S, 80S and polysome associated ribosomes was calculated and graphed as a percentage of the total area. Analysis was performed on 3 independent traces and significance (p-value < 0.05) was determined using a two-tailed t-test and is denoted by an asterisk. (**c**) Ribosomes from wild type and induced TbMTase37 RNAi cells were dissociated into free subunits in the absence of Mg^2+^ and separated through 7–27% sucrose gradients. To quantify percent reduction of 60S subunits after RNAi, the ratio of 60S to 40S was set to 1. The 60S:40S ratio for RNAi is the percent reduction compared to wild type. Error represents standard deviation of three independent replicates.

Given the nucleolar localization of TbMTase37, we explored the possibility that this protein is involved in some aspect of rRNA maturation, stability and/or ribosome function. We purified total RNA from RNAi-induced and uninduced cells. Comparison of these two fractions revealed a reduced level of one of the small LSU rRNA bands, fragment srRNA 4, in the RNAi-induced sample. In light of these results we examined other cellular RNAs, including tRNA and rRNA by Northern hybridization (Fig. 4a). An RNA unrelated to cytoplasmic ribosomes, spliced leader RNA (SL RNA), was used as a loading control for signal normalization. In this experiment RNA samples were purified in triplicate from cells after 6 days of RNAi induction, when the growth and cytokinesis phenotypes first become evident. We found that although different increases in the steady-state levels of some tRNAs were observed when compared to wild-type conditions, there was only astatistically significant (p < 0.05) reduction in srRNA 4 and increase of srRNA 2 when comparing uninduced and induced conditions (Fig. 4b). Statistical analysis was performed using one sample t-test calculating the average normalized ratio of uninduced to RNAi-induced signal for each RNA compared to the expected value of 1.0 (given that each band is at a one to one stoichiometry in the functional wild-type ribosome). To study this further, the levels of 40S, 60S, 80S and polysome-associated ribosomes in wild-type and induced TbMTase37 RNAi cell lines were compared (Fig. 5a). Upon TbMTase37 depletion, the abundance of polysomes is reduced, 80S particles accumulate, and free 60S particles decrease. Gel electrophoretic analysis of rRNAs from the gradient fractions showed under-representation of srRNA 4, particularly in the 60S and 80S fractions (Fig. 5a). Notably, we do not detect “half-mer” peaks characteristic of cells with initiation defects. To determine the extent of increases in 80S ribosomes and decrease in polysome associated ribosomes we quantified the area under the polysome traces (Fig. 5b). Three independent polysome fractionation experiments were performed and are quantified using ImageJ. Quantification revealed a significant increase in 80S ribosomes, 74% more, and a significant decrease in polysome associated ribosomes, 27% less.

**Figure 6:**
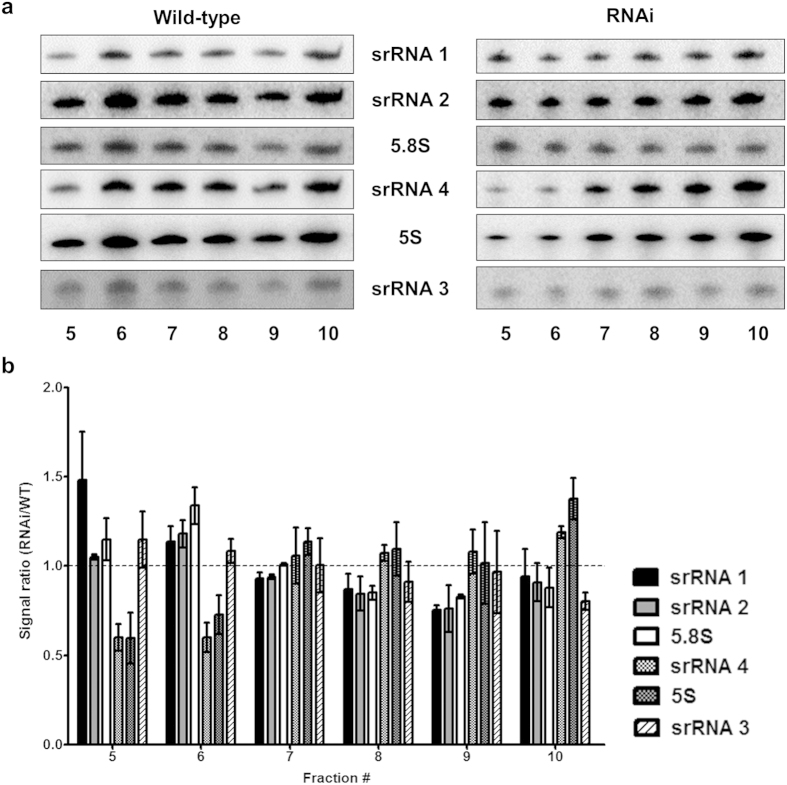
Down-regulation of TbMTase37 leads to a reduction in the steady-state levels of srRNA 4 and 5S rRNAs. (**a**) RNA isolated from the sucrose gradient fraction as in Fig. 5 were analyzed by Northern hybridization with oligonucleotide probes specific for each small ribosomal RNA fragment. Cropped panels are the signals of expected rRNA size and were used in quantification. Gels were loaded with equal A_260_ units. Fraction numbers are as in the previous figure. (**b**) Quantification of the signals in (**a**), the signal of each rRNA in each fraction was first normalized as a percentage of total probe intensity across all fractions to exclude oligo hybridization differences. Relative signals were used to calculate the signal ratio of wild type to RNAi. Calculations were made as the percentage of signal for each rRNA in each fraction divided by the sum of all probe percentages in that fraction and multiplied by 6 (the total number of rRNA bands). The dashed line across each graph represents the expected 1:1 stoichiometry for all rRNAs within a given fraction.

We next evaluated the individual rRNA species present in each sucrose gradient fraction after a second round of ultracentrifugation to pellet ribosomes complexes or subunits. RNA was extracted and analyzed by Northern hybridization (Fig. 6a). Oligonucleotide probes specific for the LSU rRNA species reveal a consistent ~1:1 ratio of all small rRNA species for wild-type cells (Fig. 6b). This trend, however, changes for TbMTase37 RNAi where levels of srRNA 4 are significantly underrepresented (p < 0.05) and 5S rRNA levels are substantially underrepresented in 60S and 80S fractions (Fractions 5 and 6 in Fig. 6), but present at nearly stoichiometric levels in polysome-associated fractions (Fractions 7–10 in Fig. 6). Notably, since srRNA4 is part of a polycistronic transcript with srRNAs 1, 2, and 3 the disproportionally low levels of srRNA 4 upon RNAi must be due to enhanced degradation after transcription. In addition, there is higher variability in relative abundance of the rRNA species after RNAi induction, consistent with incompletely assembled particles and/or decreased subunit stability. Together these results show that TbMTase37 plays an important role in maintaining the stability of the 60S subunit, as subunits lacking srRNA 4 accumulate in the absence of MTase37.

### Loss of TbMTase37 has no obvious effect on rRNA processing

**Figure 7:**
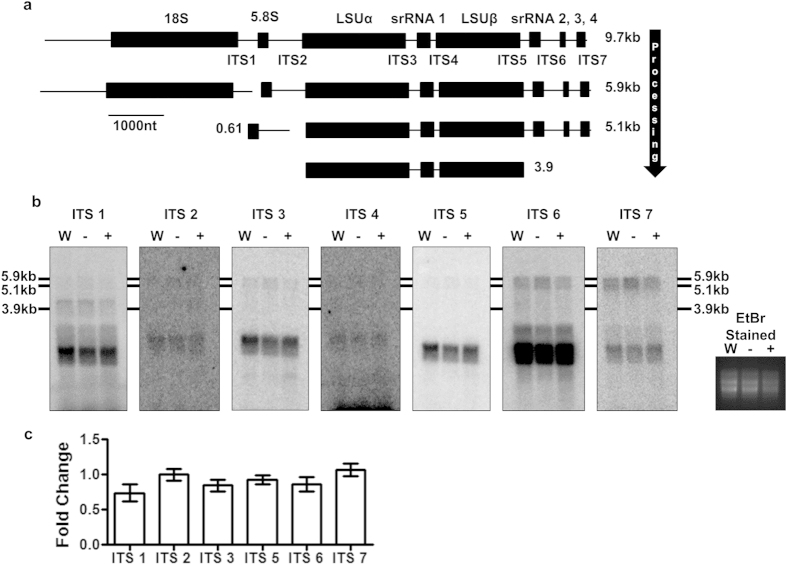
Reduction in TbMTase37 levels has no effect on pre-rRNA processing. (**a**) Schematic of *T. brucei* rRNA processing from a polycistronic transcription unit into final mature rRNAs. The names of each mature rRNA in the operon are designated with “srRNA” representing small rRNA. The lengths of predominant intermediates are specified in kilobases. (**b**) Northern blot analysis for internal transcribed spacers using RNA from wildtype (W), uninduced (−) and induced (+) cells. Formaldehyde agarose gels were loaded with equal A_260_ units and the ethidium bromide stained gel with the three large rRNA species visible (rightmost panel) shows equal RNA loading. Oligonucleotide probes specific for each internal transcribed spacer (ITS) were used to detect possible aberrant processing on full-length membranes. (**c**) The levels of 6 of the 7 ITS regions were analyzed by quantitative RT-PCR after RNAi treatment and plotted as a fold change from wild-type. All analyses were performed in triplicate. Bars represent the average change in replicates and error bars are standard error of mean. Fold change was calculated as described in Materials and Methods.

Next, we analyzed the subunit profiles for both wild type and TbMTase37 RNAi cells by lysis and sucrose gradient sedimentation in the absence Mg^2+^ to induce 80S dissociation into 40S and 60S subunits without dissociation of individual subunits into free RNA and protein (Fig. 5c)^25^. Analysis of the resulting A_254_ traces reveals a 40% reduction in 60S subunits as compared with wild type ribosomes (where the LSU:SSU ratio is set to 1 for the wild type sample). We reasoned that this could fundamentally be due to a defect in 60S stability or to a defect in LSU rRNA processing and cannot simply be caused by the lack of Mg^2+^ because wild-type ribosomes which were used for standardization were not affected by the removal of Mg^2+^ (Fig. S4). To address this question, RNA from wild type, un-induced and induced TbMTase37 cells were again analyzed by Northern hybridization using oligonucleotide probes specific for each of the 7 intergenic transcribed spacers (ITS) of the 35S pre-rRNA transcript (Fig. 7b). We observed no aberrant processing intermediates for any of our ITS probes. In case low abundance intermediates escaped detection using Northern hybridization, we also performed qRT-PCR for 6 of the spacers and again observed no significant change in ITS levels between induced and wild-type cells (Fig. 7c). Together these experiments suggest that TbMTase37 functions independently of the processing of the precursor transcript.

## Discussion

In this study we have characterized a putative methyltransferase from *T. brucei* and demonstrated its importance in the stability of the large ribosomal subunit and cell division. Although ribosome biogenesis is an essential and highly conserved process, striking differences are observed in several organisms. In trypanosomatids and *Euglena*, the large subunit 28S rRNA is processed into 6 or 13 smaller rRNA fragments, respectively^26^,^27^. Recent cryo-electron microscopy experiments showed that the *T. brucei* ribosome assembles into the canonical ribosome structure, while still possessing rRNA expansion segments and protein extensions that form a kinetoplastid-specific domain (KSD). In this structure, 4 novel intersubunit bridges are evident, including the strongest bridge (B_Tb_-3) which is mediated by the srRNA 4 fragment in conjunction with SSU protein ES12S^6^. Predictably, this ribosome may require additional rRNA processing enzymes and assembly factors. Novel chemical modifications to RNA and protein extensions can also not be excluded. A number of essential 60S ribosome maturation factors have been recently identified in *T. brucei*; all are unique to the kinetoplastid family of organisms^7^,^8^,^9^,^10^,^11^,^12^. We believe that TbMTase37 is another factor essential for ribosome function. Although most, if not all, eukaryotes possess a 3-methylcytosine methyltransferase for position 32 of specific tRNAs, many eukaryotes encode two putative paralogs of this protein. While in *T. brucei* one of the methyltransferase paralogs (TbTrm140) is indeed a tRNA methylase, no detectable activity on tRNA could be ascribed to TbMTase37 (M. Rubio, I. Fleming, K. Anderson, J.D. Alfonzo, unpublished data) and its *in vivo* target remains an open question. Significantly, the cellular target of the duplicated gene is not known in any eukaryote but the importance of this protein in cells is becoming clear. A recent report showed that METTL6 (the human homolog of TbMTase37) is one of the factors leading to cell proliferation in a subset of breast cancers via gene amplification and over-expression of the protein^20^. It would be interesting to see if, similar to the *T. brucei* situation, METTL6 achieves this effect in cell proliferation by affecting ribosome levels, in this case by increasing translational output while modifying a factor purposely limiting or destabilized in normal cells.

Our data suggest that an enzymatic activity of TbMTase37, rather than a binding activity, is important for ribosome stability and/or assembly. Experiments to detect TbMTase37 migration through sucrose gradients with assembling ribosomes showed no co-sedimentation beyond free protein. This is in contrast to Nog1 of *T. brucei*, which migrated from 60S subunits to the end of the gradient as previously reported (Fig. S3)^10^, possibly ruling out a direct role in ribosome assembly. We tried to address the importance of TbMTase37’s conserved catalytic domain by creating a catalytically dead variant of this gene with a mutated SAM-binding domain; however, several attempts at *T. brucei* transformations failed suggesting, but not proving, that this version of the protein may exert a dominant negative effect. Notably, recombinant expression systems are notoriously leaky in *T. brucei* and even in the absence of induction the catalytic mutant may be expressed at sufficient levels leading to toxicity.

Experiments to separate ribosomes and single subunits revealed reduced polysome-associated ribosomes and a significant decrease in 60S subunits. While there has been a report of functional ribosomes lacking a single rRNA, there are no reports of translationally competent ribosomes with sub-stoichiometric levels of multiple rRNA species^28^,^29^. It is interesting that following RNAi, polysome-associated ribosomes from sucrose gradient fractions possess all rRNAs (including all fragments) in equimolar amounts, but corresponding 60S and 80S fractions specifically lack the srRNA 4 band in comparison to wild type. The presence of lower levels of srRNA 4 in these fractions likely reflects a combination of relatively long ribosome half-life and the incomplete nature of RNAi knockdowns and may speak to the essentiality of TbMTase37. We could not detect an accumulation of expected processing intermediates supporting the fact that the absence of srRNA 4 in the 60S and 80S fractions was not a result of defects in pre-rRNA processing. The only obvious defect was a decrease in srRNA 4 (one of the 6 small rRNA fragments) and, to a lesser extent, 5S rRNA, which may be a secondary effect of the destabilized ribosomes. We also observed an increase in the levels of some tRNAs (Fig. 4) but are unsure of the significance of changes in specific tRNA levels. We do not expect the observed differences in hybridization to be due to loss in modification since uninduced and induced samples exhibit no significant change in levels and the ethidium bromide stained gel clearly shows the increase in tRNA sized RNAs. Perhaps the increase is a stress related transcriptional response to the depletion of MTase37 and lower levels of functional ribosomes. Interestingly, in cells depleted for Nop1, a nucleolar protein with a methyltransferase domain and connections to rRNA processing and ribosome assembly, tRNA methylation was significantly increased^30^. The possibility that tRNA transcription led to higher methylation cannot be ruled out based on the published data. We were unable to detect changes in the modification set of total tRNA population after MTase37 depletion but the connection with tRNA abundance and/or methylation would be a fascinating aspect to study further.

Since we are able to detect an increase in 80S complexes in RNAi induced cells, it seems that the defects in ribosome biogenesis due to lack of TbMTase37 do not prevent the partial assembly of ribosomes lacking LSU srRNA 4. However, these “defective” 80S ribosomes are unable to engage in translation elongation as shown by a striking decrease in polysome-associated ribosomes. This suggests that beyond its role in forming a key inter-subunit bridge shown in the recent cryo-EM structure, srRNA 4 plays a critical function in maintaining the integrity of the 60S particle, which may result in an inactive 80S initiation complex in an otherwise fully assembled ribosome. These observations also raise the important question of how these defective particles are turned over via quality control pathways, while revealing a level of malleability in trypanosome ribosome biogenesis. In other eukaryotic systems, turnover of nonfunctional ribosomes is a proteasome-dependent process. Prior to degradation of nonfunctional ribosome, subunits must first be dissociated in a Cdc48 complex-dependent manner^31^. *T. brucei* encodes a Cdc48 homolog which has not been extensively characterized and studies on the 20S proteasome show divergence in substrate specificity from the human complex lending the possibility that pathways for ribosome turnover differs in trypanosomes^32^,^33^.

The lack of srRNA 4 and 5S rRNA in non-translating assembled ribosomes suggests TbMTase37 is imparting stability to assembled ribosomes since loss of this protein does not obstruct assembly. Typically the 5S rRNA is assembled into the 66S pre-ribosome in the nucleolus as a ribonucleoprotein particle together with proteins L18 and L5. Cryo-EM experiments from yeast have demonstrated that the 5S particle associates with the assembling ribosome in an orientation 180° rotated from its final confirmation and must be turned to stabilize the structure prior to assembly^34^. In fact, two trypanosome specific proteins, p34 and p37, are shown to be essential factors in 5S rRNA abundance and early assembly into the pre-ribosome cooperatively with ribosomal protein L5^7^,^35^. In RNAi experiments against p34 and p37 cells exhibited similar morphological changes to those described in this work supporting the connection with ribosome stability and rRNA abundance^7^. It is possible that *T. brucei* srRNA 4 is required to be present for proper assembly of 5S rRNA into the ribosome or to induce a conformational change.

Although we did not observe pre-rRNA processing defects the putative methylation activity of TbMTase37 may be modifying one or more rRNAs prior to or during processing which affect the proper assembly and/or stability of a fully formed ribosome. A modification may be targeted to srRNA 4 or interacting rRNAs, which provides an essential docking point for interaction and assembly with other ribosomal RNAs or proteins. It was demonstrated in *S. cerevisiae* that Nep1 is required for small subunit biogenesis as both a biogenesis factor and rRNA methyltransferase forming m^1^acp^3^ψ at U1191^36^,^37^. Bud23p, a m^7^G methyltransferase, is known to play a role in processing of 18S rRNA and is thought to participate in disassembly of the SSU processome prior to export of pre-40S ribosomes^38^. Although we did not observe TbMTase37 migration through sucrose gradients in a large complex, it is possible TbMTase37 plays a role similar to Bud23p in directing methylation to processed rRNAs and induces disassembly of a sub-processome complex. Along these lines a DEAH-box helicase, Dhr1, was recently shown necessary to dissociate U3 snoRNA from pre-rRNA to permit folding of the central pseudoknot^39^.

In cells depleted for TbMTase37, at the onset of the slow growth phenotype (day 6), we observed increases in cell size and dysregulation of progressive cell division despite being unable to observe clear cell cycle defects when measuring cellular DNA content by flow cytometry (data not shown). TbMTase37 strictly localizing within the nucleolus suggests that any modification produced by TbMTase37 may be important for long-term stability of some of the rRNA small fragments (e.g. srRNA 4 and 5S) and the observed phenotype is the result of either decreased ribosomal rRNA half-life or decreased large subunit stability. Loss of TbMTase37 and a decrease in functional ribosomes likely induces nucleolar stress. In other eukaryotic systems depletion of ribosomal proteins or impairments to rRNA transcription and processing generates nucleolar stress and elicits both a p53 dependent and independent responses^15^. *T. brucei* lacks the p53 protein and many other well-characterized cell cycle control proteins. The putative human methyltransferase METTL6 was recently identified as a key genetic driver or regulator of oncogenesis that is essential for cell proliferation^20^. Sequence alignment of TbMTase37 and human METTL6 reveals a high degree of similarity and conserved methyltransferase domain (Fig. S1). Our data for MTase37 correlates well with predicted function and published importance of METTL6 in cell proliferation. Consequently, control of mitosis checkpoints and transition from mitosis to cytokinesis in this highly diverged eukaryote must have evolved novel approaches to coordinate these essential processes.

## Materials and Methods

### Strains, Plasmids, and Cell culture

For TbMTase37 RNAi, a portion of the coding sequence (Tb.927.9.11750) was amplified by PCR from *T. brucei* 29-13 gDNA and cloned between BamHI and HindIII sites of the p2T7-177 vector^21^. The TbMTase37 expression plasmid was constructed but introducing the entire coding sequence into the BamHI and HindIII sites of pLew79^40^. The resulting construct was linearized using NotI for integration during electroporation of procyclic *T. brucei* 29-13 cells. All growth was performed in SDM-79 supplemented with phleomycin at 27 °C. Clones were selected over a period of 14 days and then tested for growth phenotype or expression by addition of tetracycline at 1μg/ml to growth media. Growth was monitored every 24h using a hemocytometer. The most responsive clone was selected for all subsequent experiments.

### Western blot analysis

Polyclonal antibody for TbMTase37 was prepared by rabbit immunization performed with 4 subcutaneous injections of 0.5mg purified recombinant TbMTase37 emulsified with complete (injection 1) and incomplete (injections 2–4) Freund’s adjuvant. Specific TbMTase37 polyclonal antibody was isolated by affinity purification from rabbit serum using immobilized recombinant TbMTase37. Cell lysates corresponding to 5 × 10^6^ cells/well were separated on 15% SDS–polyacrylamide gel, blotted, and probed. The polyclonal rabbit antibodies against TbMTase37, Isd11^41^, Nog1^10^ and enolase (kindly provided by P.A.M. Michels) were used at 1:100, 1:1.000, 1:1000 and 1:1.000 dilutions, respectively. Secondary anti-rabbit IgG antibodies (1:6000) coupled to horseradish peroxidase (GE Healthcare) were visualized according to the manufacturer’s protocol using the ECL kit (Pierce).

### Northern blot analysis

RNA was isolated using guanidinium thiocyanate/phenol/chloroform extraction method^42^. For analyses of small rRNA bands 10 μg of cellular RNA was separated on denaturing 8% polyacrylamide/8M urea gel, electroblotted to Zeta-probe membranes and cross-linked to the membrane with UV light. For analysis of RNA bands greater than 1 kb, 3 μg of cellular RNA was separated on 1% agarose-formaldehyde gel and transferred to Zeta-Probe membrane by capillary transfer. Northern blotting was performed using 5′-^32^P labeled oligonucleotide probes according to manufacturer’s instructions (Bio-Rad). Images were obtained and quantified using Typhoon FLA 9000 and ImageQuant TL software (GE Healthcare). All RNA was visualized by staining with ethidium bromide.

### Oligonucleotide probes

Sequences for oligonucleotide probes used in this study are listed in supplementary information.

### Sucrose density gradient sedimentation

For polysome profile analysis, 50 mL shaking cultures were grown overnight at 27 °C to mid-log phase. Cyclohexamide was added to cultures at 100 μg/mL final and incubated at 27 °C for an additional 10 minutes. Cells were immediately chilled and collected by centrifugation. All subsequent steps were carried out at 4 °C. Cells were washed twice in 15 mL of ice-cold PBS supplemented with 100 μg/mL cyclohexamide and final pellet suspended in 790 μL of polysome buffer (10 mM Tris-HCl pH 7.5, 300 mM KCl, 10 mM MgCl_2_) supplemented with 100 μg/mL cyclohexamide, 2 mM DTT, 1X Protease inhibitor cocktail and 20 units of RNase inhibitor. Cells were lysed by addition of NP-40 to a final concentration of 0.25% and incubated on ice for 5 minutes. Lysates were clarified by centrifugation at 15,000 *g* for 10 minutes. Three milligrams equivalent of OD_260_ units was loaded on each 10–50% sucrose gradient prepared in lysis buffer and centrifuged at 36,600 RPM for 2 hours at 4 °C in a Beckman SW41Ti rotor. A gradient fractionator (ISCO UA-6 UV Vis with Type 11 optical unit) was used to record UV profiles and fractions were collected manually for subsequent analysis. Subunit profile analysis was performed as described above except MgCl_2_ excluded from buffers and 0.5 mg of RNA was centrifuged on a 7–27% sucrose gradient for 3.5 hours.

### Nuclei fractionation

Nuclei were purified using a method adapted from Shapiro and Doxsey^43^. Briefly, log phase cells were washed in PBS and suspended at 5 × 10^8^ cells/ml in 1 mM PIPES pH7.4, 1 mM CaCl_2_, 500 mM Hexylene glycol, 125 mM sucrose. Cells were broken by passage through Stansted homogenizer at 20PSI and monitored for breakage by microscopy. Cellular structures were pelleted, suspended in 5 mL of supernatant and separated at 60,000 × *g* for 35 minutes through a 35% Percoll gradient with 1 mM PIPES pH 7.4, 5 mM CaCl_2_, 500 mM Hexylene glycol and 700 mM sucrose. The bottom most band containing pure nuclei was collected by side puncture, washed in the same buffer as the gradient and suspended at a concentrated volume for analyses.

### Quantitative RT-PCR

RNA was first isolated as described for Northern blot analysis above and subsequently DNase treated with RQ1 DNase according to manufacturer’s instructions. qRT-PCR was performed using One Step SYBR^®^ PrimeScript™ RT-PCR Kit II (Takara) according to manufacturer’s instructions with 0.4 μM oligo and 10 ng of RNA per reaction. *trm140* and *isd11* mRNAs were used as internal controls. Data analysis was completed using the comparative C_T_ method as described previously^44^.

### Immunofluorescence microscopy

For experiments with kinetoplast staining, MitoTracker^®^ Red was added to the culture at 500 nM and shaken at 27 °C for 30 minutes to allow uptake. Cells were harvested for microscopy at 2–5 × 10^6^cells/mL and washed twice in PBS before suspending in 3.7% formaldehyde/PBS solution and 100 μl of suspension was fixed to glass slides for 10 minutes. Cells were washed three times in PBS, permeabilized with 0.1% Triton X-100 and washed an additional three times in PBS. Blocking was performed for 60 minutes using 5.5% FBS supplemented with 0.05% Tween 20 in PBS. All incubation steps were performed in humid chamber and all antibodies were used at concentrations indicated in text. Cells were stained using 100 uL 1 μg/mL DAPI solution for 1 minute and washed three times with PBS. Cells were air dried and mounted using Vectashield^®^ before imaging was performed using a Nikon Ti microscope. Image analysis was performed using ImageJ (NIH) and Nikon Elements AR.

## Additional Information

**How to cite this article**: Fleming, I. M.C. *et al.* A tRNA methyltransferase paralog is important for ribosome stability and cell division in *Trypanosoma brucei*. *Sci. Rep.* **6**, 21438; doi: 10.1038/srep21438 (2016).

## Supplementary Material

Supplementary Information
