# Supplementary material for: A tRNA methyltransferase paralog is important for ribosome stability and cell division in Trypanosoma brucei
Source: Sci Rep. 2016 Feb 18;6:21438. doi: 10.1038/srep21438 (PMC4757839; doi:10.1038/srep21438)
Supplement: Supplementary Information [file srep21438-s1.pdf]

A tRNA methyltransferase paralog is important for ribosome stability and cell division in *Trypanosoma brucei*

Ian M.C. Fleming[1], Zdeněk Paris[1,4], Kirk W. Gaston[2,5], R. Balakrishnan[1,2], Kurt Fredrick[1,3], Mary Anne T. Rubio[1] and Juan D. Alfonzo[1,3,*]

Supplementary Material

Supplementary Figure 1. Sequence alignment of the two *T. brucei* paralogs (TbTRM140 and TbMTase37) with TRM140 of *Saccharomyces cerevisiae*. Black boxes (*) indicate identical amino acids shared among the sequences, while amino acids highlighted in gray (: or .) denote conservative changes. The putative SAM-binding domain is indicated by the black filled rectangle labeled "AdoMet Binding Domain" and it is conserved in all four proteins.

Supplementary Figure 2. Specificity of His antibody for immunofluorescent microscopy. DIC represents light microscope view of wild-type *T. brucei* cells. DAPI (blue) was used to detect the nuclear (N) and mitochondrial kDNA (K). Fixed cells were mixed with α-His primary antibody and fluorescently-labeled secondary antibody (488nm) for imaging background binding of His antibody in cells.

Supplementary Figure 3. Co-migration of proteins with ribosome intermediates. Western blot analysis of sucrose gradient fractions from polysome fractionation experiments. Gradient fractions were concentrated by centrifugation at high speed and separated by SDS-PAGE. Nog1 and TbMTase37 were detected through gradient fractions using antibodies specific for each protein. Fractions are labeled 1-17 and "in" denotes total cell input of gradient.

Supplementary Figure 4. Subunit rRNA composition does not change after salt-free dissociation. Wild-type polysome associated ribosomes were purified by sucrose gradient centrifugation and pelleted for analysis. A portion of the sample was suspended in salt-free polysome buffer to induce subunit dissociation and subunits were separated by a second round of gradient centrifugation. The rRNA composition in the presence (first centrifugation) or absence (second centrifugation) of 10mM $^{Mg2+}$ was analyzed by Northern blot. An oligonucleotide specific for srRNA 1 was used as a standardization to show that srRNA 4 levels do not differ between the two salt conditions.

Supplementary Table 1. Oligonucleotides used in this study.

Target	Sequence (5'-3')
ITS1	CATCTCCCATGTCAAACGGC
ITS2	CAACACACAAATCCACAC
ITS3	CTGTCCCACATGCAATTTC
ITS4	CTCACACACACATGGCTATTC
ITS5	GCACATGCCACCGAACGGC
ITS6	CAAGCCGTGCCCACACAC
ITS7	GTAATGAAATTCTTCCGTTGC
srRNA-1	GATGGGTCACCTGGCAAGTG
srRNA-2	CCATACTTCCCTCACGATGC
5.8S rRNA	CACTTTGCTGCGTTCTTCAACG
srRNA-4	GCATGTACTCTCGTTTGGAGAG
5S rRNA	GGTGCATTCGGCCAAGTATGG
srRNA-3	GCAGAGCCCACCAGATAAGAG
Spliced Leader	GCTGCTACTGGGAGCTTCTCATAC
12S rRNA	AGGAGAGTAGGACTTGCCCT
tRNA Thr (858)	GGATCGAACCCCTGACCTCCG
tRNA Ile (3119)	CCAACAGGGGTCGAACCTGTGAC
tRNA His (3585)	GGAATCGAACCCGGGTATT
tRNA Leu (3586)	AACCCACGCCTCCGGAGAG
tRNA Pro (3587)	GGAATTGAACCCGGGACCT
Tbm3A RNAi (3129)	GATCCAGGATCCTGGAGCAGGAGAAGTTTACTGCCTGG
Tbm3A RNAi (3130)	AGCAGTCAAGCTTATTAGACCGCTGCCTCTGCGTACGCG

```
TbMTase37   ---MPDKIENNTRKR-------------------------------PRELPFVEDYRPYT 26
TbTrm140    -MSFSSDEKKPTRTRGGGALTDPHA---VKLHGNDFSLDEKLGGLAPEERKVVAEYIEKC 56
ScTrm140    LKALTQDVKEETLENIAHEGRGDNTGDQNAVEKSDFEKSDTEGSRIGRDLPFEFGKRNLT 348
               :... :: *  .                                .:  .

TbMTase37   GG--------------------QLAQLKVR--KTTHKEHWDQYYRNN-----TLNGYRDR 59
TbTrm140    EE--------------------TLLQLSKRGCNTPIIPFNSENHSWD-----SLYKVNKR 91
ScTrm140    EESDVWDHNAWDNVEWGEEQVQQAEEKIKEQFKHPVPEFDKKLYNENPARYWDIFYKNNK 408
                                     :   .  : .   . .: :  :      :   ..:

TbMTase37   HYILREFSELREALERLKKNNEATLEECVWMEAGCGVGNAVFPILKDYGDVSGWRVVGFD 119
TbTrm140    HFPLKNYIILAFPLLKSICCG-PKRESKYIVECGCGTGSTLLPIMNQFKD--GVHFIGFD 148
ScTrm140    ENFFKDRKWLQIEFPILYASTRKDAEPVTIFEIGCGAGNTFFPILKDNEN-ENLRIIAAD 467
            .  :::   *   :           *    .* ***.*.:.:**:::  :  . :.:. *
                              AdoMet Binding Domain

TbMTase37   ISTVAIKLIREKQNTLPHVEQEKFTAWVLNPVEQDIAPIITAS-------------VARQ 166
TbTrm140    VSTAAVSALLEHPIASDFSAEGRLTVFPYDLCYGRVSASEDCRRTKFKTECGTLKTTLLE 208
ScTrm140    FAPRAVELVKNSEQFNPKYG--HATVWDLANPDGNLPDGVEPH----------------- 508
            .:. *:. : :           : *.:        :.
                              AdoMet Binding Domain

TbMTase37   SRPRGDGLVDFVSMIFVLCSIP-VEEHAVVLRRIAACMKEGGVFFFRDYCVDDHAEKRFS 225
TbTrm140    KVPGCSKGVDAAILVFVLSSLPTIECMLYALTEIKSILHNDGILLFRDYAVPDHNLFRFV 268
ScTrm140    -------SVDIAVMIFVFSALA-PNQWDQAMDNLHKILKPGGKIIFRDYGAYDLTQVRFK 560
                    ** . ::**:.::.  :    .: .:   :: .* ::**** . *    **
                              AdoMet Binding Domain

TbMTase37   AHCRVEAN--TESRTNGTLSHFFSVSELRDLFCSVGFELIN---VEVVEREVVNRREGMN 280
TbTrm140    RQQNKKHNDLSFCKGDGTLQMFFELNFTRKLFALAGLKEVEGHGLQYHCNRIVNRKNSKR 328
ScTrm140    KNRILEEN--FYVRGDGTRVYFFSEEKLREIFTKKYFLENK---IGTDRRLLVNRKRQLK 615
             :   : *   : : :**   **. .  *.:*    :   :   :    . :***:.  .

TbMTase37   LQRRFLQGRFRKIRRIGDNGIDIAGNGNLSRNMPQK 316
TbTrm140    MDKIFINGSFCLS----------------------- 341
ScTrm140    MYRCWVQAVFDVPQ---------------------- 629
            : : :::. *
```

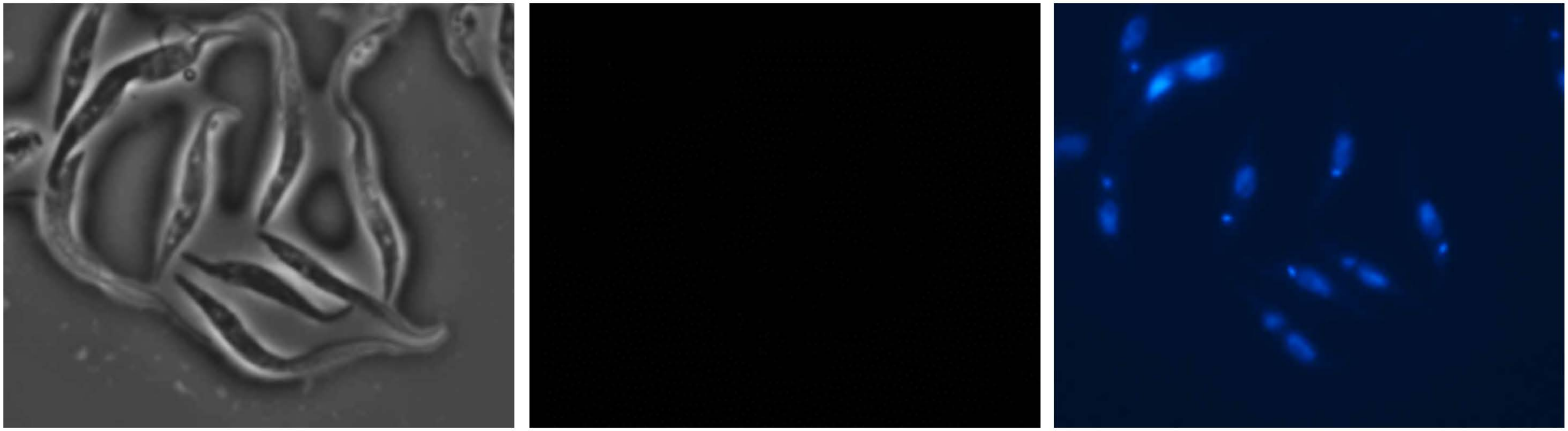

DIC α-His DAPI

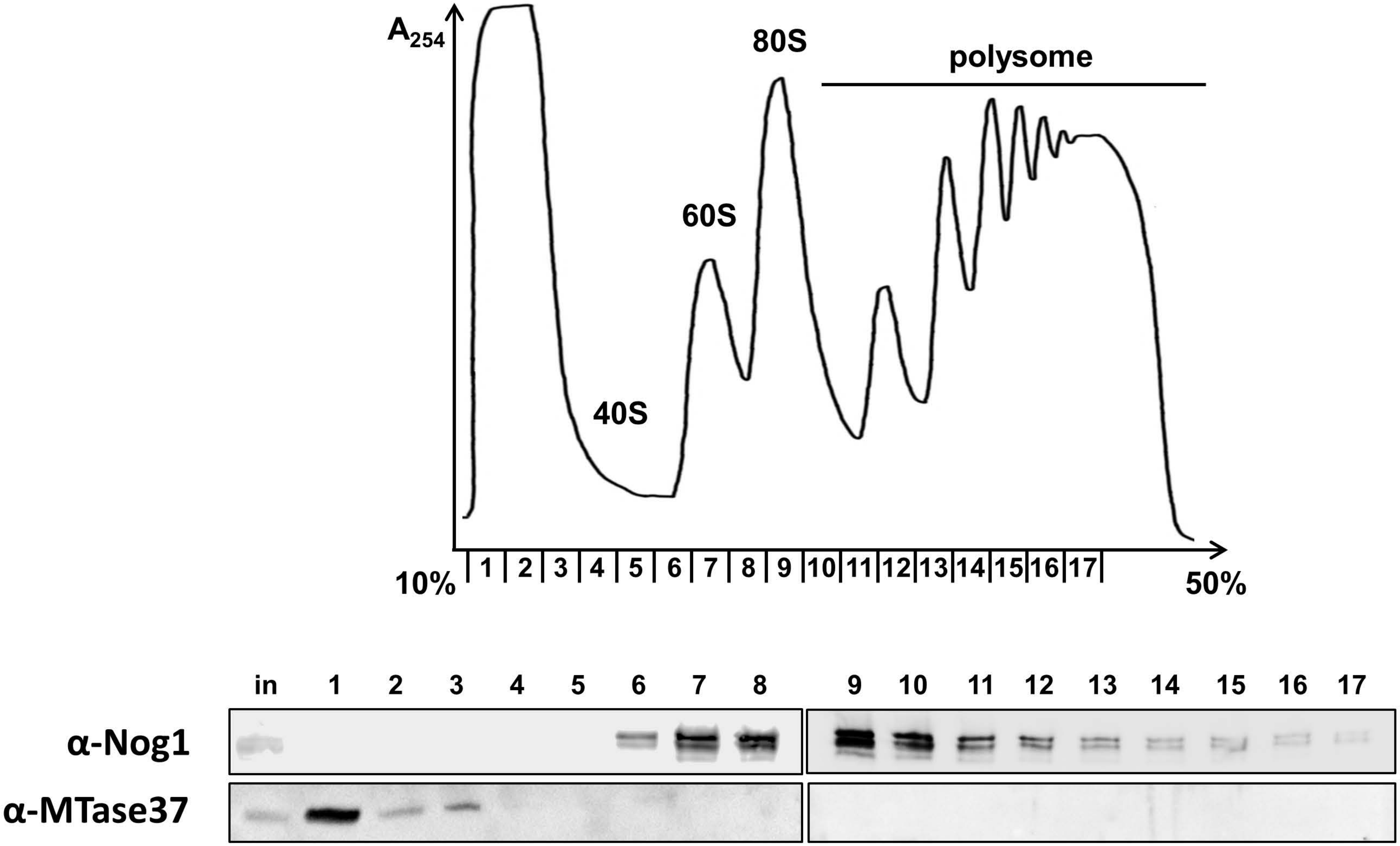

A254
80S
polysome
60S
40S
1 2 3 4 5 6 7 8 9 10 11 12 13 14 15 16 17
10%
50%
in 1 2 3 4 5 6 7 8 9 10 11 12 13 14 15 16 17
α-Nog1
α-MTase37

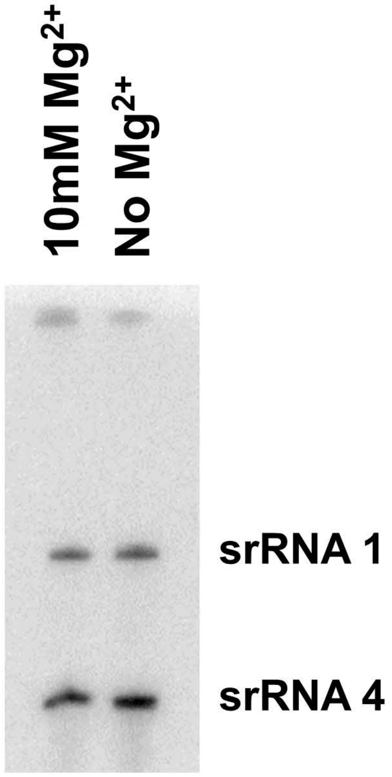
10mM Mg^{2+}
No Mg^{2+}
srRNA 1
srRNA 4
